# The role of protein hydrolysates for exercise-induced skeletal muscle recovery and adaptation: a current perspective

**DOI:** 10.1186/s12986-021-00574-z

**Published:** 2021-04-21

**Authors:** Paul T. Morgan, Leigh Breen

**Affiliations:** School of Sport, Exercise and Rehabilitation Sciences, University of Birmingham, Edgbaston, Birmingham, B15 2TT UK

**Keywords:** Concentrates, Hydrolysates, Isolates, Muscle protein anabolism, Protein synthesis, Supplementation

## Abstract

The protein supplement industry is expanding rapidly and estimated to have a multi-billion market worth. Recent research has centred on understanding how the manufacturing processes of protein supplements may impact muscle recovery and remodeling. The hydrolysed forms of protein undergo a further heating extraction process during production which may contribute to amino acids (AA) appearing in circulation at a slightly quicker rate, or greater amplitude, than the intact form. Whilst the relative significance of the rate of aminoacidemia to muscle protein synthesis is debated, it has been suggested that protein hydrolysates, potentially through the more rapid delivery and higher proportion of di-, tri- and smaller oligo-peptides into circulation, are superior to intact non-hydrolysed proteins and free AAs in promoting skeletal muscle protein remodeling and recovery. However, despite these claims, there is currently insufficient evidence to support superior muscle anabolic properties compared with intact non-hydrolysed proteins and/or free AA controls. Further research is warranted with appropriate protein controls, particularly in populations consuming insufficient amounts of protein, to support and/or refute an important muscle anabolic role of protein hydrolysates. The primary purpose of this review is to provide the reader with a current perspective on the potential anabolic effects of protein hydrolysates in individuals wishing to optimise recovery from, and maximise adaptation to, exercise training.

## Introduction

One of the most debated topics in sports, exercise, and health nutrition is the importance of dietary protein, with a host of studies investigating different types, amounts and timings of protein ingestion on exercise-induced skeletal muscle adaptation [1, 2]. Recent research has centered on understanding how the manufacturing processes of protein supplements may influence muscle anabolism, recovery and remodeling [3]. For example, protein hydrolysates are produced by chemically unfolding proteins and enzymatically hydrolysing the peptide bonds at various points in its primary structure to produce varying quantities of shorter chain peptides and free amino acids (AA) [4]. The treatment of proteins in this way essentially results in *‘pre-digestion’*, which has been suggested to facilitate subsequent AA absorption, resulting in a more rapid increase in circulating AAs [5]. Protein supplements are often used to support the building and maintenance of muscle proteins, with studies suggesting the branched chain amino acid (BCAA) leucine is of particular importance for the stimulation of postprandial muscle protein synthesis (MPS) through mechanistic target of rapamycin complex1 (mTORC1) signaling [6, 7]. The consumption of whey protein, for example, is characterised by accelerated AA absorption kinetics, resulting in the rapid appearance of leucine into circulation [8–12].

The stimulation of MPS is driven primarily by essential amino acids (EAA) and appears to be triggered by leucine [7, 13–19], in a dose-dependent manner at rest [20, 21] and postexercise [22], up to EAA and protein intakes of ~ 10 and ~ 20 g (0.18–0.30 g·kg^−1^), respectively, in young adults [20–22]. The postprandial MPS response is further augmented by prior exercise, with greater postprandial MPS responses observed up to about 24 h after cessation of an exercise task [23]. However, the postprandial increase in MPS is transient and returns to basal rates after ~ 1–2 h even in the presence of elevated plasma AA concentrations, a phenomenon referred to as the ‘muscle full effect’ [24, 25]. This might suggest that high concentrations and more rapid rates of AA appearance is not optimal for net protein balance, thus questioning the importance of AA digestion and absorption kinetics [26–28]. In support of this notion, there are a number of reports that do not support a role for modulating AA appearance rate on MPS [26–28]. Indeed, dietary AAs provided at levels and/or rates that are in excess of their ability to be incorporated into new proteins results in their deamination [29]. Nevertheless, it has been suggested that the muscle anabolic potential of a given protein may be influenced or dictated by the digestion and absorption kinetics [6, 8]. Indeed, the presence of more favourable AA digestion and availability for elevated MPS stimulation with protein ingestion has also been observed [30], and is discussed in more detail below.

The purpose of this review is to provide an overview of the current understanding of protein hydrolysates, which may contribute to a more rapid increase in circulating AA concentrations and AA delivery to the muscle [3], with a particular focus on the impacts on skeletal muscle anabolism. Much of the focus in this review is on healthy individuals wishing to optimise recovery from, and maximise adaptation to, exercise training. However, given the sparse nature of this research field, data is presented from several models and population groups, to provide a broad overview of the use of protein hydrolysates on human metabolism. We discuss any theoretical basis of the use of protein hydrolysates and its future applications.

### Potential for protein production strategies?

Despite previous studies not supporting a role for modulating AA appearance rate on postprandial MPS [26–28], there has been recent interest into the role of AA appearance rate on MPS and intramuscular anabolic signaling in the postexercise period [31–33]. It has been suggested that the magnitude and rate of aminoacidemia (particularly of leucine) is the primary nutritional stimulus for postexercise MPS [8, 31, 34], and this is termed the ‘leucine trigger hypothesis’ [35]. Furthermore, the post-exercise MPS response to whey protein ingestion is attenuated when ingested in multiple smaller doses over time versus bolus feeding [31]. These data suggest that protein digestion and absorption kinetics, and timing of intake, may modulate the MPS response even when AA composition is matched. However, a number of other studies assessing postexercise protein ingestion have failed to support a role of aminoacidemia distinct from the impact of protein dose in regulating postexercise MPS [26, 36–39]. Nevertheless, the capacity of protein to stimulate MPS has been suggested by others to be dependent on both its AA profile as well as its digestion and AA absorption kinetics [8, 40]. Indeed, the latter two are the main components of dietary protein quality indexes such as the Protein Digestibility Corrected AA Score (PDCAAS) and Digestible Indispensable AA Score (DIAAS), which is the current preferred method for differentiating between protein sources due to its more accurate ability to measure AA absorption [40, 41].

The discrepant findings on the muscle anabolic properties of comparably high-quality protein sources (i.e., whey and casein) may be explained by the total protein (and EAA) content of the ingested proteins, the time frame of MPS assessment [7, 36, 42, 43] or the manufacturing process of the protein. To the latter, as an example, studies have used calcium caseinate to detect differences in the muscle anabolic properties of whey and casein proteins [36, 38, 43]. This is pertinent to note as micellar casein undergoes additional acidification to produce calcium caseinate (a less structured form of micellar casein), which alters the digestion kinetics, such that the rate of plasma AA appearance more closely mimics whey [36, 38, 43, 44]. However, by contrast, a recent study has found that calcium caseinate was digested and absorbed more slowly when compared with micellar casein, which could be due to the additive effect of calcium (as opposed to sodium) on clotting [44]. It is also reasonable to suggest that any additive specific processing strategies as well as the storage of such proteins (i.e., via glycation) may also impact the rate of digestion and absorption [44, 45].

Despite recent studies not supporting the rate of aminoacidemia as an important factor influencing postprandial and postexercise MPS rates, there is growing interest in how the protein supplement manufacturing process may influence skeletal muscle anabolism [3]. It has long been suggested that protein hydrolysates, providing a higher proportion of di-, tri- and smaller oligo-peptides, are superior for muscle growth compared with intact proteins and free AAs [3, 46]. Due to a higher proportion of shorter chain peptides, protein hydrolysates may exhibit faster absorption and digestion rates, and thus, attenuating the sequestering of AAs by the splanchnic bed and rapidly increase plasma AA concentrations and intracellular leucine accumulation [3, 47, 48]. There is also a growing interest in plant-based proteins as a means to reduce in the intake of animal foods whilst maintaining sufficient protein intake for whole-body health [49, 50]. However, plant-based proteins typically contain a lower content of leucine (typically < 7%) compared with animal-derived proteins (typically > 10%), as well as an incomplete EAA profile [51, 52]. Furthermore, plant-derived proteins generally display lower digestibility, ultimately leading to inferior muscle anabolic effects compared with dose-matched animal-derived proteins [53–56], though recent work has challenged this notion, suggesting that there are no differences in MPS rates following ingestion of a dose-matched (30 g) wheat protein, milk protein, or a protein blend of milk and wheat, at least in healthy young males [57]. Though, it is pertinent to note that the participants recruited in this study were likely highly sensitive to anabolic stimuli given their age and health status and that a 30 g dose of relatively lower-quality protein was sufficient at maximising the acute muscle anabolic response [57]. Undoubtedly, isolated plant-derived proteins may only support maximal muscle anabolism when consumed in considerably high amounts and/or in combination with other suitable plant-derived proteins to achieve a full complement of EAAs. Whilst food fortification techniques have been discussed elsewhere [51, 58], it may therefore be prudent to consider how alterations in processing strategies may modulate the digestibility/absorption of lower-quality protein sources and the bioavailability of AAs, which are known to impact the muscle anabolic potential of proteins [51, 52, 58]. Finally, most work assessing the postprandial MPS response has focused on isolated protein intake, yet dietary protein is typically consumed as part of a mixed meal. Whether the digestion and absorption rate of a protein, and/or the co-ingestion of other nutrients, modulates the MPS response when relatively large doses of protein are ingested and when MPS is assessed over prolonged (i.e., > 6 h) free-living periods remains to be determined.

## Protein hydrolysates and bioactive peptides

Protein supplements are available predominantly in powdered forms and undergo a number of manufacturing processes during production. For example, in its whole form, bovine (cow’s) milk contains ~ 20% whey, with the remaining 80% from casein protein [59]. Following the addition of acid (or enzymes) to heated milk, casein coagulates and separates from the remaining liquid substance (whey) [60]. These substances are then washed and dried into a powdered form for use in food products/supplements. The following section will discuss, briefly, the manufacturing process of protein isolates/concentrates and hydrolysates and introduce bioactive peptides. However, the step-by-step discussion of the specific production process of each protein source is beyond the scope of this narrative, but discussed in detail elsewhere [61–63].

### Protein concentrates and isolates

Concentrates are considered the most basic form of protein supplement, requiring minimal additional processing, and typically containing small quantities of other nutrients (e.g., fats and carbohydrates) and represent the lowest cost option [64]. As a result, protein concentrates can vary greatly in their protein content between suppliers (containing anywhere between ~ 30 and 90% protein, but typically ~ 70–80% protein) [64]. Some may prefer concentrates as they contain the naturally occurring nutrients derived from the manufacturing process, such as immunoglobulins for milk-derived proteins, that *may* enhance immune function and reduce oxidative stress [65, 66]. By contrast, protein isolates are refined in a process that minimises extraneous carbohydrates (i.e., lactose for milk-derived proteins) and fats, producing a compound of > 90% protein content [64].

### Protein hydrolysates

Protein hydrolysates are a concentrate or isolate that has undergone several purification steps, in which some of the peptide bonds are broken by exposure to additional heat, acids or proteolytic enzymes, producing large quantities of free AAs and shorter chain peptides different lengths (i.e., di-,tri- and smaller oligo-peptides) [61, 63, 67, 68]. The hydrolysis of proteins can be achieved by the use of single or multiple enzymes, the choice of which depends on the protein source and required degree of hydrolysis [61]. Following hydrolysis, the product is evaporated, pasteurised, and dried [61]. Whilst the method of acid hydrolysis offers the advantage of low cost, this process results in the complete loss of tryptophan, partial loss of methionine, and the conversion of asparagine into aspartate and of glutamine into glutamate [67]. By contrast, enzymatic hydrolysis represents a higher-cost option but provides more mild conditions (i.e., temperature and pH) for hydrolysis and thus does not result in any loss of AAs and/or compounded by the existence of residual chemicals within the product [61]. Further, proteases provide more precision for controlling the degree of peptide-bond hydrolysis [61]. The relative proportion of di-, tri- and oligo-peptides within a given compound are determined by the degree of hydrolysis and thus, the percentage of cleaved peptide bonds [61]. The proportions of free AAs, smaller and larger peptides within a protein hydrolysate will vary according to a number of additional factors including; the source of protein, the quality of water and the type of proteases [61]. Following ingestion of intact dietary proteins, proteins (i.e., folded polypeptide chains) are broken down into their constituent AAs and/or smaller peptides (i.e., di-, tri- and oligo-peptides), absorbed by the intestine and transported in the blood prior to absorption/utilisation in the body [69]. Further, in the small intestine, large peptides are hydrolysed to small peptides, which are absorbed into enterocytes faster than free AAs into the circulation [61]. Thus, with a higher relative proportion of shorter chain peptides, it is suggested that protein hydrolysates are more readily digested and absorbed and thus increase circulating AA concentrations more rapidly than ‘intact’ proteins [61, 70, 71], with suggestions that this process enhances AA bioavailability and MPS stimulation (discussed below) [72]. Similar to isolates, hydrolysates can also be beneficial for individuals with lactose sensitivities (to milk-derived proteins) but have the added advantage of being easier to consume for those who suffer with additional digestive problems [72,72].

### Bioactive peptides

Bioactive peptides are produced in larger quantities following protein hydrolysis [61, 62]. Interestingly, there may be other reported benefits to protein hydrolysates (i.e., cardiovascular, nervous, immune, gastrointestinal) through the delivery of these bioactive peptides which are released during hydrolysis [61, 62, 73, 74]. Bioactive peptides are defined as the fragments of AA sequences in a protein that provide biological functions beyond their nutritional value [75]. They have been suggested to exert cholesterol-lowering, antidiabetic, antithrombotic, antihypertensive, anti-cancer and antimicrobial effects [62]. Further, these peptides have also been linked with anti-obesity and antioxidant effects [61], as well as support insulin secretion [76]. A previous review has also summarised the impacts of protein hydrolysates and its bioactive peptides on tissue repair, post-surgical and severe burn recovery, gastric repair and pressure ulcer recovery [5]. Bioactive peptides usually contain 2–20 AA residues and are activated following release by hydrolysis during processing and/or gastrointestinal digestion [61, 77]. In recent years, numerous bioactive peptides have been identified as being present or generated from various protein sources [61]. Arguably, the most extensively studied bioactive peptides has been on the tri-peptides Val-Pro-Pro (VPP) and Ile-Pro-Pro (IPP) which have been shown to elicit antihypertensive effects via angiotensin I-converting enzyme (ACE) inhibitory activity [61, 62]. Indeed, there is evidence that the hydrolysis of many proteins generate potent ACE inhibitors including milk [78], meat [79] and eggs [80]. However, undoubtedly further research on the role of bioactive peptides is required to fully understand their potential long-term in vivo health effects in humans. Indeed, a particularly interesting avenue of research might be to investigate the potential of protein hydrolysates and the associated bioactive peptides in older individuals who exhibit chronic low-grade inflammation given its multiple purported health benefits.

## Digestion and absorption kinetics

AA composition and digestive properties can vary greatly between different isolated types of intact proteins, protein blends (i.e., a combination of isolated proteins) and different forms of the same protein source [2], the latter potentially via alterations during the manufacturing process. Therefore, the latter will be the focus of the following section, with specific reference to the digestion and absorption kinetics of protein hydrolysates. Due to the requirement of a sophisticated triple isotope tracer approach to truly assess postprandial protein digestion and absorption kinetics [81–83], many studies have used serial plasma AA concentrations in the postprandial period following the consumption of a protein bolus to infer differences in digestion and absorption characteristics of protein sources. It is therefore important to note that in the following section, changes in postprandial plasma AA concentrations (i.e., aminoacidemia or AA responses), where appropriate, are used as a proxy for interpreting differences in intestinal absorption rates. Nonetheless, we do acknowledge that this is not without contention [81, 82]. We recognise that the use of serial plasma AA concentrations in isolation provide limited insight into the flux of AAs from the splanchnic region as well as the contribution from endogenous AA release [84]. Indeed, some exogenous AAs may be incorporated into splanchnic tissues upon first pass [85, 86]. The use of intrinsically isotopic labelled proteins will help address these important concerns.

### Protein digestion and absorption

An in-depth discussion of protein digestion and absorption can be viewed elsewhere [41, 87–89]. Protein digestion is the process of breaking down proteins into smaller fragments. Subsequent absorption refers to the process of uptake of these smaller fragments (i.e., AAs, di-, tri- and smaller oligo-peptides) from the gastrointestinal lumen to support skeletal muscle anabolism [41, 61, 69]. Importantly, as the capacity of the gastrointestinal lumen to release AAs is inferior to the capacity of the small intestinal to absorb AAs, protein digestion typically represents the limiting step in AA availability [41]. Once protein-rich foods have been chewed and swallowed, the process of protein digestion starts with chemical breakdown by gastric acid and pepsin within the stomach [87]. Following gastric emptying, the protein is delivered in the duodenum of the small intestine, whereby pancreatic enzymes further facilitate hydrolysis [88, 90]. Subsequently, AAs, di-, tri- and smaller oligo-peptides are released and taken up across the intestinal mucosa [89]. Whilst a small portion of these absorbed AAs then undergo uptake within splanchnic regions, the majority of absorbed AAs are released into circulation for the use by peripheral tissues [2, 91]. The fraction of ingested protein that is not digested and absorbed in the small intestine reaches the large intestine where AAs are deaminated and metabolised by microbiota [92, 93].

### Protein hydrolysates: digestion and absorption kinetics

It has been suggested that protein sources containing a higher proportion of di-, tri- and oligo-peptides are more rapidly absorbed than those with longer peptide chains, due to the requirement of additional hydrolysis prior to absorption of intact proteins [47, 69–71, 94, 95] and this may result in significantly greater increases in plasma concentrations AAs and shorter-chain peptides [71]. Although one may expect free AAs to be more rapidly digested and absorbed, the speed of enzymatic peptide hydrolysis and H^+^-dependent di- and tri-peptide transporters in the small intestine ensures polypeptides achieve plasma aminoacidemia with a similar latency as free AA ingestion [61]. The available evidence on whether hydrolysed proteins digest and appear in circulation more rapidly than non-hydrolysed proteins is equivocal (discussed below). This lack of consensus might be explained at least in-part, by the degree of hydrolysis of a protein source, which is infrequently reported in studies investigating the muscle anabolic effects of hydrolysed proteins. It is also pertinent to note that studies of AA kinetics rarely provide detail on how hydrolysis was achieved which may have important implications for the interpretations that can be drawn. The following section will summarise the main studies to date that have attempted to assess the differences in digestion and absorption kinetics between different protein sources/forms in young and older adults, rodent models, as well as discuss the potential for hydrolysis to augment the muscle anabolic properties of lower-quality plant-derived proteins.

### Young adults

To investigate the potential of protein hydrolysis to favourably alter AA digestion/absorption kinetics, Power et al., (2009) compared a 45 g bolus of whey protein isolate to a dose-matched whey hydrolysate, independent of carbohydrate [96]. However, in this study, Power et al., (2009) failed to identify any differences in the rate of AA appearance, assessed via plasma AA concentrations at rest [96]. Specifically, whilst the rate of gastric emptying was more rapid with the hydrolysate, statistical analysis revealed that the estimated rate of gastric emptying was not altered by hydrolysis of the protein (18 vs. 23 min, P = 0.15) [96]. However, maximum plasma insulin concentration was 28% greater following the ingestion of the whey protein hydrolysate, presumably due to mechanisms beyond gastric emptying [96]. Indeed, a recent study has also investigated the effects of carbohydrate supplemented with a moderate dose of sodium caseinate protein or a sodium caseinate protein hydrolysate (0.16 g·kg^−1^) following prolonged aerobic exercise in young trained male cyclists [97]. The hydrolysate was associated with an augmented insulin and anabolic signaling response (discussed below), however, this did not alter the plasma AA profile, which was also reported to be similar following hydrolysis of a whey protein isolate [98]. Therefore, the mechanism responsible for augmented insulin secretion seems to be independent, at least in part, of altered AA kinetics. One potential explanation for the insulinotropic action observed with protein hydrolysates may be the increased provision of bioactive peptides (discussed briefly above), however this warrants further investigation [76]. Together, these findings suggest that protein hydrolysates may induce a more potent insulinotropic effect, which may be important for muscle protein anabolism given the permissive effects of insulin on MPS when sufficient AAs are present, as well as its influence on inhibiting muscle protein breakdown [99]. Though it is worthy of note that, whilst feeding reduces muscle protein breakdown rates via an increase in circulating plasma insulin concentrations, only a moderate rise in insulin concentration is required for maximal inhibition of muscle breakdown rates [99].

Such findings are also in agreement with Calbet & Holst (2004) who observed statistically similar gastric emptying (assessed via tritiated water) and plasma AA responses to complete/intact and hydrolysed whey protein sources at rest (Gastric emptying rate: 21.3 vs. 19.3 vs. 19.4 min for whey hydrolysate, casein hydrolysate and whole whey protein, respectively) [100]. Though it is acknowledged that the lack of statistically significant differences was with the exception of a whole (non-hydrolysed) casein protein for which the speed of intestinal AA absorption was significantly slower than its hydrolysed form (18.0 min). Whilst these findings may reflect the true lack of difference in digestion and absorption kinetics between the whey protein hydrolysate and its non-hydrolysed form, they may also be explained by fasted-state conditions. Specifically, the digestion of AAs would be expected to be more rapid on an empty stomach and without the co-ingestion of other macronutrients (i.e., higher splanchnic uptake) [96, 101]. Alternatively, as whey protein is considered a rapidly absorbed protein with high bioavailability (> 95%) even in its intact non-hydrolysed form, it may be that hydrolysis fails to significantly enhance absorption kinetics [8, 102]. This opens up the notion that hydrolysis may not be beneficial for high-quality protein sources and, instead, may offer more interest as a potential avenue to optimise the digestion and absorption kinetics slower digested proteins (i.e., micellar casein) and/or lower-quality (i.e., plant-derived) protein sources, the latter which is discussed below.

Comparative studies investigating protein digestion rates have demonstrated more rapid AA kinetics with hydrolysed proteins compared with whole cows’ milk at rest [103]. Specifically, Calbet and MacLean (2002) compared the differences in plasma AA concentrations following nitrogen-matched bolus’ of a pea protein hydrolysate, whey peptide hydrolysate and a cow's milk solution containing complete milk proteins [103]. Whilst this study did not assess protein-matched controls (i.e., non-hydrolysed forms), the peptide hydrolysates (i.e., pea and whey) elicited a faster increase in plasma AA concentration when compared with the milk solution. Interestingly, in agreement with the studies discussed above [96, 100] and despite the higher carbohydrate content of the milk solution, the peptide hydrolysates elicited a peak insulin response that was 2–4 times greater than that evoked by than the milk solution. Further, the insulin response was closely associated with the plasma AA response (particularly BCAAs), regardless of the rate of gastric emptying [103]. Though it is worthy of note that in this study the treatments displayed differences in energy–density and this might explain, at least in part, some of these discrepant observations (921 vs. 963 vs. 2763 kJ/L for pea, whey and milk protein, respectively) [103].

Moro et al. (2019) speculated that consumption of a whey protein hydrolysate compared with a whey protein isolate would induce greater postprandial intracellular leucine accumulation [48]. To test this hypothesis, in a dual-tracer study design to assess MPS (discussed below) and AA kinetics, participants received a low dose (0.08 g·kg^−1^ of body weight) of either a whey protein hydrolysate or an intact (non-hydrolysed) whey protein mixture at rest. However, no significant differences in the plasma concentrations of BCAAs (i.e., valine, leucine, isoleucine) were found. The authors also found that the rate of leucine transport into muscle (assessed via stable isotope tracer of L-[1-^13^C]-leucine) was significantly higher with the whey protein hydrolysate, which resulted in a slightly higher muscle intracellular concentration of leucine over 3 h after ingestion [48]. However, it is important to note that AA transport may also have been enhanced by the proton-dependent oligo-peptide transporter which transports shorter chain peptides [48, 104]. Such findings on total BCAA plasma levels are in contrast with Morifuji et al., (2010) who found a higher plasma concentration of BCAA with protein hydrolysates compared with the intact (non-hydrolysed) form at rest [71]. The authors speculated that this discrepancy could be explained by the total amount of protein ingested in the 2 studies (Moro: 6 g protein, 1.4 g BCAA vs. Morifuji: 12 g protein, 2.8 g BCAA), which could slow the rate of appearance of plasma AAs in the latter [48, 71]. In addition, despite the expected differences in whey vs. soy proteins in this study, similar observations to the whey protein hydrolysate compared with its intact form were found with soy protein hydrolysate compared with the non-hydrolysed form [71]. Importantly, the whey and soy protein hydrolysates led to significant increases in the concentrations (and aminoacidemia) of the dipeptides Val-Leu and Ile-Leu compared with non-hydrolysed proteins [71].

To further understand the potential for protein hydrolysis to positively influence plasma AA appearance, a recent study compared three different whey protein hydrolysates with varying degrees of hydrolysis (23, 27 and 48% of cleaved peptide bonds, containing 11, 15 and 35% di- and tri-peptides, respectively), via plasma AA concentrations at rest [102]. The authors demonstrated superior plasma AA appearance rates with a whey protein hydrolysate compared with an intact casein protein control. However, the degree of hydrolysis did not significantly impact total AA appearance, at least within the given ranges of hydrolysis assessed [102], potentially due to the small differences in di- and tri-peptide content between the hydrolysates and/or the endogenous enzymatic hydrolysis in the gut, which may override the initial differences in the degree of hydrolysis to ultimately produce similar absorption rates [102]. However, a general non-significant trend was reported for greater peak plasma leucine, total leucine (0.0111 vs. 0.0076 vs. 0.0072 vs. 0.0024 mol L^−1^·min^−1^, for high, medium, low degree of hydrolysis and casein, respectively) and total EAA (0.0384 vs. 0.0338 vs. 0.0331 vs. 0.0114 mol L^−1^·min^−1^, for high, medium, low degree of hydrolysis and casein, respectively) appearance with the highest degree of protein hydrolysis. Furthermore, the highest PDCAAS also corresponded with the highest degree of hydrolysis for the branched chain AAs leucine (High: 264 ± 1.5, Medium: 136 ± 0.5, Low: 161 ± 1.2), isoleucine (High: 201 ± 1.2, Medium: 181 ± 0.7, Low: 182 ± 1.3) and valine (High: 177 ± 1.0, Medium: 124 ± 0.5, Low: 130 ± 0.9), respectively [102].

One study has found a whey protein hydrolysate (25 g dose) to be inferior at elevating plasma AA concentrations when compared with a protein-matched whey protein isolate at rest [105]. Specifically, whilst the hydrolysed whey resulted in similar total plasma AA appearance 60 min post ingestion, thereafter, AA content declined rapidly while AA concentrations for the isolate remained elevated for a further 45 min [105]. The authors suggested that the impaired AA availability observed with the hydrolysate might be explained by the processing procedures (i.e., heat, alkaline treatments) producing compounds (i.e., d-amino acids, lysinoalanine) that may impair digestibility and bioavailability of the protein [102, 106].

### Older adults

Despite whey exhibiting a high AA bioavailability, the splanchnic extraction of AAs has a critical influence on their availability to peripheral tissues and therefore on whole body protein metabolism [107]. This is pertinent to note as, in theory, under conditions where splanchnic extraction is increased (i.e., aging), the use of a protein hydrolysate may lead to favourable effects on bioavailability with potential implications for skeletal muscle anabolism [34, 86, 91, 107–109]. Further, it is plausible to suggest that in older adults, who are less sensitive to an anabolic stimulus (i.e., a protein bolus) and have been reported to consume insufficient per meal amounts of protein to maximise MPS [110, 111], the use of protein hydrolysates may be beneficial to speed up the digestion and absorption of AAs (and particularly leucine) to initiate MPS and support net muscle protein balance [35]. Though studies assessing digestion and absorption capabilities of protein hydrolysates in older adults are limited, a 20 g dose of intrinsically–labelled hydrolysed casein protein has been shown to result in peak plasma insulin, leucine and EAA concentrations in older men at rest that are more comparable with whey protein than the intact form of casein [8] and such findings may have important implications for the older adult. Further, over a 6-h postprandial period, exogenous phenylalanine appearance rate was 27% higher (Peak phenylalanine appearance rates during the first 105 min: 0.92 ± 0.03 vs. 0.79 ± 0.04 µmol·phenylalanine·kg^−1^· min^−1^, respectively) and splanchnic extraction significantly lower (Casein hydrolysate: 66.1 ± 1.2 vs. Casein: 73.0 ± 1.4%) after ingestion of a 35 g dose of hydrolysed casein protein compared with an intact casein protein (L-[1-^13^C]-phenylalanine-labelled) in older adults at rest [70]. Plasma AA concentrations also increased to a greater extent (25–50%) after the ingestion of the hydrolysate (assessed via multiple intravenous AA tracers). Peak insulin concentrations were significantly higher with the hydrolysate (Casein hydrolysate: 50.2 ± 7.6 vs. Casein: 26.2 ± 3.7 mU/L) [70]. Together, these data demonstrate that, at least in a more slowly digested protein, hydrolysis has the capacity to accelerate protein digestion and absorption from the gut and reduce splanchnic extraction in older adults, with potential implications for skeletal muscle anabolism [8, 70]. Ultimately, more research is warranted on the use of protein hydrolysates in older adults, particularly given the relative importance in maintaining muscle mass in older age [112].

### Rodent studies

Comparative studies investigating protein digestion and absorption rates in rodents have demonstrated more rapid AA kinetics with hydrolysed protein compared with free AAs of equivalent content [113, 114]. Similarly, Roberts et al., (2014) has demonstrated that a moderately hydrolysed whey protein (15–20% degree of hydrolysis) elicits a more rapid postprandial increase in plasma AAs compared with a whey protein concentrate [115]. Specifically, whilst both conditions increased plasma AAs (1.2–2.8-fold), BCAAs (1.2–1.7-fold), and serum di-, tri- and oligo-peptides (1.1–2.7-fold) 60 min post-ingestion, the whey protein hydrolysate increased lysine and tended to increase isoleucine and valine further. However, despite the apparent consistent superior impact of protein hydrolysates on rates of protein digestion and absorption in rodents, the findings may not readily translate to humans [116].

### Lower-quality proteins

Alterations of lower-quality proteins during production is of interest as a potential avenue to enhance the AA response following consumption of proteins that are otherwise considered suboptimal to maximise net muscle protein balance compared with higher quality proteins at comparative doses [53]. It is plausible that lower-quality plant-derived proteins, as a result of slower digestion and absorption, may also result in greater splanchnic extraction, and this provides further justification for investigating the potential of hydrolysis in plant-derived proteins [51, 117]. For example, a greater rate of AA oxidation following the ingestion of 40 g of soy protein has been observed compared with the ingestion of a matched dose of whey protein [118]. Studies using isotope tracer methodology to measure whole body utilization of AAs have also shown a higher deamination of AAs derived from wheat (25% of ingested nitrogen deaminated) than milk (16%) protein within 8 h of consumption in healthy individuals [119].

In addition to the study discussed above demonstrating superior effects of soy protein hydrolysates compared with the non-hydrolysed form [71], a recent study has shown that hydrolysis of a lower-quality plant protein blend had no impact on the plasma EAA response, when assessed using plasma AA concentrations at rest [117]. Specifically, Brennan et al., (2019) found that hydrolysis of a 34 g dose of a plant-blend (pea and pumpkin hydrolysate) failed to enhance EAA bioavailability over a 4 h postprandial period when compared with: 1) a pea and pumpkin isolate (34 g); 2) a pea, pumpkin, sunflower and coconut blend (33 g); and 3) a whey protein isolate control (24 g), which were matched for total leucine and EAA content [117]. Indeed, plasma AA appearance with the whey protein isolate was significantly elevated compared with the plant-blends. This suggests that other factors beyond EAA (and leucine) content, such as an increased proportion of AAs being sequestered in the splanchnic region with plant vs. animal proteins [51, 117], influence the reduced appearance of plasma AAs with plant-derived proteins. The lack of enhanced AA appearance with the hydrolysed plant-blend was surprising but may be explained, in-part, by the moderate degree of hydrolysis (< 15%), such that a higher percentage of cleaved peptide bonds may have driven a significant rise in the digestion/absorption of AAs and subsequent anabolic effects [117]. Interestingly, and in contrast to these observations, techniques such as heat treatment combined with hydrolysis, could significantly reduce trypsin inhibitory activity in plant-derived proteins, which are known to impair digestibility, and thus enhance the bioavailability of plant-derived proteins and overall nutritional value [53, 106, 117, 120–122].

In another study, Gorissen et al., (2016) found that despite a lower EAA content, a wheat protein hydrolysate (35 g; containing 2.5 g of leucine) was similarly digested and absorbed (postprandial increase in plasma EAA concentrations; Whey: 2.23 ± 0.07 vs. Casein: 1.53 ± 0.08 vs. Wheat: 1.50 ± 0.04 mM) when compared with micellar casein but not whey, in a study that combined plasma EAA concentrations with stable isotope methodology (continuous infusion of L-[ring-^13^C_6_]-phenylalanine) at rest [53]. Following ingestion of the wheat protein hydrolysate, plasma lysine and methionine concentrations in particular, increased only marginally when compared with casein and whey, which is in agreement with the lower lysine (1.5%) and methionine (0.6%) contents in wheat protein compared with casein (7.6 and 2.1%, respectively) and whey (10.1 and 2.0%, respectively) proteins [53]. Plasma concentrations of key EAAs did not differ between the wheat protein hydrolysate and an intact (non-hydrolysed) wheat protein [53]. In direct contrast, methionine concentrations increased to a greater extent following the ingestion of the wheat protein control when compared with the hydrolysate [53]. However, phenylalanine concentrations and appearance rates, as measured using mass spectrometry, increased to a similar extent after the ingestion of both intact and hydrolysed wheat proteins, with no differences between conditions. Ultimately, more research is required to investigate the impact of plant hydrolysates, per se, on digestion and absorption kinetics using an appropriate plant protein control. Given the impaired digestibility of plant-based proteins, hydrolysis may indeed significantly improve digestibility and bioavailability of AAs.

## Digestion and Absorption Summary

**Fig. 1:**
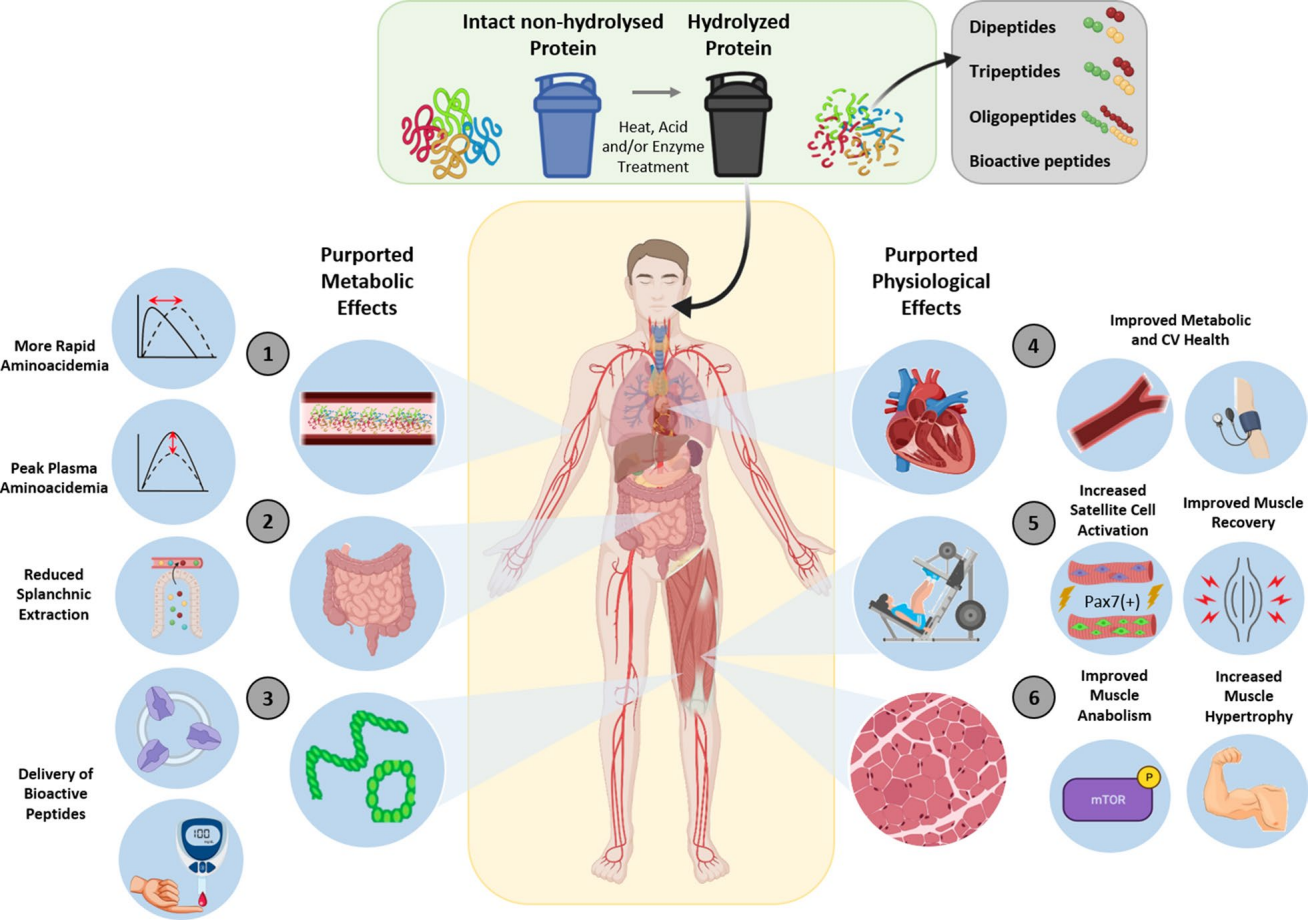
Summary of the purported biological effects of and physiological outcomes with protein hydrolysates compared with intact non-hydrolysed proteins. It has been suggested that the ingestion of a more rapidly digested and absorbed hydrolysed protein (containing larger amounts of di-, tri- and smaller oligo-peptides), may result in a more rapid and/or higher concentration of circulating amino acids (AA) [1], less efficient uptake of AAs by the splanchnic bed [2] and the delivery of a greater amount of bioactive peptides [3], thus maximising tissue AA delivery. As a result, these purposed metabolic effects may lead to improved metabolic, CV and general health outcomes [4], increased satellite cell activation/proliferation and improved recovery from exercise (via reduced muscle damage) [5] and/or enhanced muscle anabolism and muscle hypertrophy [6]

It is plausible that protein hydrolysates may increase the rate of digestion and absorption of AAs. However, there is currently limited evidence to support and/or refute such claims. There is, though, potentially interesting applications of protein hydrolysates into older adults and on lower-quality proteins that are largely unexplored. Much of the available data provide limited insight into the flux of AAs from the splanchnic region and undoubtedly, further well-designed studies with appropriate protein controls and incorporating intrinsically labelled proteins, tracer infusions and serial blood sampling are required to quantitatively assess the impact of the protein manufacturing process on AA flux and incorporation into muscle. Furthermore, liquid forms of protein are known to achieve peak concentrations of AAs twice as quickly after ingestion of solid protein-rich foods that also typically contain more slowly digestible carbohydrates and dietary fiber [123], which compose the majority of an individual’s diet, and should be considered in future research on the digestion and absorption capabilities of protein hydrolysates. Indeed, a greater delay in protein digestion and absorption kinetics can be expected following a typical mixed meal [33, 124]. Exercise may shift the sensitivity of the muscle to AA stimulation [23], however, there is a near absence of studies investigating AA profiles when combined with exercise following protein hydrolysate consumption. It is pertinent to note that, although somewhat speculative, we cannot at this stage discount a potential impact of exercise on AA digestion/absorption kinetics with hydrolysed proteins, as AA digestion has been shown to be altered with exercise [2, 33, 125]. However, it is unlikely that completing exercise prior to ingestion of a protein source changes the pattern of delivery of EAAs that would differentiate between different protein sources [123]. Figure 1 provides a summary of the purported metabolic and physiological effects of protein hydrolysates.

## Skeletal muscle anabolism and exercise-induced adaptation and recovery

Though we acknowledge the effects of hydrolysates on digestion and absorption kinetics is equivocal, on the basis of a potentially favourable increase in EAA appearance rates with hydrolysed proteins, the following section aims to provide a summary of the current literature which has investigated the potency of hydrolysed protein to impact: (1) skeletal muscle anabolism (MPS and anabolic signaling); and (2) components of exercise-induced adaptation and recovery.

### MPS and anabolic signaling in young adults

More rapid digestion and absorption kinetics of proteins may have a greater effect on MPS, exercise-induced MPS and muscle mass accretion [7, 70, 71]. The paucity of studies investigating the differences in hydrolysates vs. isolates have found some interesting results in young adults, albeit with a predominant focus on hydrolysed whey over other protein sources. For example, Moro et al., (2018) investigated the muscle anabolic potency of a small dose of whey protein hydrolysate (0.08 g·kg^−1^) on mTORC1-mediated signaling and MPS in healthy young men at rest [48]. The authors found that, when compared with an intact whey protein (matched for total protein content), no differences in the phosphorylation of S6K1 (a marker of mTORC1 activation for MPS) were observed (increased equally by ∼20% 1 h post ingestion) [48]. However, whilst phenylalanine utilization for synthesis, assessed via phenylalanine infusion (L-[ring-^13^C_6_]-phenylalanine), increased above fasted values at 1 h post-ingestion with both protein supplements to a similar extent (~ 43% increase in mixed-muscle MPS at rest), the response remained elevated 3 h post-ingestion only for the hydrolysate condition despite muscle protein breakdown being similar between treatments, suggesting that the hydrolysate may possess superior muscle anabolic capabilities [48]. Nevertheless, anabolic potency for promoting overall MPS across a 3-h time frame was similar between protein sources (Isolate: + 57% vs. Hydrolysate: + 67% above basal values). Whilst some of these findings are of interest, they are somewhat surprising given the proposed mechanism for protein hydrolysis on speeding AA kinetics and the subsequent muscle anabolic response, as opposed to the delayed superior anabolic effects observed in this study [48].

A moderate dose of sodium caseinate protein or a sodium caseinate protein hydrolysate (0.16 g·kg^−1^) combined with carbohydrate has been shown to enhance intracellular anabolic signaling (phospho 4E-BP1 Thr^37/46^) when compared with a carbohydrate isocaloric control after prolonged aerobic exercise in young trained cyclists, a response which was further augmented with the protein hydrolysate [97]. However, in this context it is important to consider the sometimes discordant relationship between the phosphorylation of anabolic intracellular targets and measured rates of MPS [24, 99, 126]. Finally, it is pertinent to note that, although somewhat speculative, we cannot at this stage discount a potential impact of exercise on postprandial MPS. Indeed exercise may shift the sensitivity of the muscle to AA stimulation and therefore slight differences between protein sources may be magnified following exercise compared with at rest [23].

### MPS and anabolic signaling in Older adults

There is currently limited data investigating the muscle anabolic effects of protein hydrolysates in older adults at rest and/or combined with exercise. However, a ~ 30% increase in 6-h postprandial mixed-muscle MPS has been observed following the consumption of a 35 g dose of casein hydrolysate compared with a casein isolate in healthy older adults at rest (Casein: 0.054 ± 0.004 vs. Casein hydrolysate: 0.068 ± 0.006%·h^−1^) [70]. This is an interesting finding in this population given the relatively high dose of protein administered (35 g) to maximise MPS. It is possible that the rapid absorption and greater systemic appearance of AAs following the casein hydrolysate consumption could explain the superior muscle anabolic response compared with a naturally slow-release casein protein in its intact form that, as discussed above, could be particularly potent in the older adult which exhibit muscle anabolic resistance and increased splanchnic AA retention (particularly leucine) leading to attenuated aminoacidemia [86, 127]. These findings of a more slowly digested protein may also have important implications for whole-foods which are typically consumed with other macronutrients. In addition, the delivery profile of AAs may affect the ability of a smaller protein bolus or one less abundant in leucine, to stimulate MPS, particularly in muscle less sensitive to anabolic stimuli, highlighting the importance of separate consideration of specific protein prescription in older and/or sarcopenic individuals. [13, 18, 21, 128]. In a study by Pennings et al., (2011), protein ingestion (20 g dose of whey, casein, or casein hydrolysate) was combined with continuous intravenous L-[ring-^2^H_5_]-phenylalanine infusion to assess in vivo digestion and absorption kinetics (discussed above) of these dietary proteins and its association with muscle anabolism [8]. In this study, whilst whey demonstrated superior anabolic effects (attributed to faster digestion and absorption kinetics and higher leucine content), EAA concentrations and postprandial mixed-muscle MPS rates in the casein hydrolysate (Whey: 0.15 ± 0.02 vs. Casein: 0.08 ± 0.01 vs. Casein hydrolysate: 0.10 ± 0.01%·h^−1^) were more comparable with whey protein than the intact form of casein at rest in older men [8]. Indeed, a strong positive correlation (r = 0.66) was observed between peak plasma leucine concentrations and postprandial MPS [8].

### MPS and anabolic signaling in Rodent studies

A recent study using a rodent model investigating the effects of whey protein hydrolysate (0.5 and 2.0 g·kg^−1^·day^−1^) compared with an intact whey protein on postexercise MPS has produced interesting findings with regards to the muscle anabolic potential of hydrolysates [129]. Specifically, following a 2-h swimming protocol, MPS 60 min following ingestion of the whey protein hydrolysate was significantly higher compared with a control intact whey protein. Interestingly, the whey protein hydrolysate caused greater MPS and phosphorylated 4E-binding protein 1 (4E-BP1) levels compared with the control whey protein only at the lower dose (i.e., 0.5 g·kg^−1^·day^−1^) [129], which supports the notion that whey protein hydrolysates may be more efficacious under conditions of suboptimal protein intakes and in relative proximity of exercise, albeit in rodents. Similar findings have been also observed with anabolic signaling markers and the attenuation of muscle loss with whey hydrolysates compared with its constituent AAs and/or non-hydrolysed forms [130–132].

### Lower-quality proteins and MPS

There is currently limited data exploring hydrolysed plant protein sources on MPS. Nevertheless, a recent study (discussed above on digestion and absorption kinetics) reported that postprandial MPS stimulation following the consumption of wheat protein hydrolysate (35 g) was 30–40% lower than with whey protein isolate or micellar casein (Casein: 0.050 ± 0.005 vs. Wheat: 0.032% ± 0.004%·h^−1^) in older adults at rest [53]. Unsurprisingly, whilst myofibrillar MPS rates were lower after ingesting a 35 g dose of wheat protein hydrolysate than after the same amount of casein, ingesting a larger quantity of wheat protein hydrolysate (i.e., 60 g) improved MPS rates to a level similar to the animal-derived isolate proteins (Wheat [60 g]: 0.049 ± 0.007%·h^−1^) [53]. However, whilst more effective than a lower dose of wheat protein hydrolysate (i.e., 35 g), it is imperative to note that this requires consuming a significant quantity of wheat. Although the primary purpose of the study was not to assess the impact and practicality of such large doses of plant-derived protein hydrolysis, per se, the consumption of high quantities of plant proteins may not be a feasible strategy, particularly in older adults, due to a number of potential contributing factors (i.e., appetite and appetite suppression, chewing capabilities, cost) [133–135]. Indeed, the higher dietary protein recommendations that have been suggested for older individuals to maximise MPS (~ 1.6 g·kg^−1^·day^−1^) [111], may pose a challenge for older individuals with compromised appetite, particularly given the known dose-dependent satiating effects of protein [136–140]. It is worthy of note that the digestibility and biological value of wheat protein is particularly poor compared with other plant-derived protein sources (low in the EAAs leucine, methionine and lysine) and this might explain the lack of superior anabolic effects of a plant-based protein hydrolysate at lower quantities on markers of muscle anabolism [58, 141]. Interestingly, the more sustained appearance of plasma AAs observed with the higher dose of wheat protein hydrolysate (i.e., 60 g) compared with the 35 g dose of whey protein was associated with a greater stimulation of postprandial MPS rates [53].

These findings are contrast to Pinckaers et al., (2021) who reported similar rates of postprandial myofibrillar MPS at rest following the consumption of lower doses (30 g) of a wheat protein hydrolysate compared with milk protein (Milk: 0.053 ± 0.013 vs. Wheat: 0.056 ± 0.012%∙h^−1^) [53, 57]. However, as the authors speculated, this is likely explained by the inclusion of healthy young compared with older adults [53, 57]. Indeed, the anabolic resistance that is typically observed in the older adult, likely contributed to the impaired muscle anabolic response at suboptimal doses of EAAs in the study by Gorissen et al., (2016) [110, 142–145]. Importantly, the wheat hydrolysate provided leucine (2.1 g) and EAA (8.2 g) doses that were sufficient to stimulate a robust increase in MPS in healthy young adults [57].

### MPS Summary

The impact of protein hydrolysates on MPS regulation at rest and following exercise are unclear. Indeed, there is a near absence of studies investigating MPS when combined with exercise following protein hydrolysate consumption. As such, further research with appropriate protein controls and lower-quality protein sources, particularly within relative proximity to exercise, are warranted to truly understand the muscle anabolic potential. This is particularly important in individuals who are not consuming sufficient quantities of protein to maximise net protein balance (i.e., ~ 1.6 g·kg^−1^·day^−1^), as in populations that are already consuming sufficient total dietary protein, protein supplementation and/or protein source does not seem to further contribute to skeletal muscle anabolism and adaptation [111, 146, 147]. Indeed, there is evidence to suggest that the use of protein hydrolysates may be able to provide similar postprandial aminoacidemia that would support a robust increase in MPS in proteins containing less EAA and leucine content [148].

### Exercise-induced chronic muscle adaptation and acute recovery

Much of the current available literature on the effects of protein hydrolysates on exercise-induced adaptation and recovery has focussed on young adults with consumption of whey protein. Unfortunately, we were only able to identify a small number of studies which were not confounded by the lack of appropriate non-hydrolysed protein controls [4, 97, 149, 150]. In one study, Buckley et al., (2010) found that following a bout of eccentric knee extension exercise, the consumption of 25 g of hydrolysed whey protein was associated with enhanced recovery of peak torque in sedentary males compared with a matched dose of whey protein isolate [149]. Specifically, peak isometric torque decreased by ∼23% following the eccentric exercise task and remained suppressed 6 h following the consumption of a flavoured water and whey protein isolate, yet recovered fully with the whey protein hydrolysate [149]. These findings are comparable with a follow up study by the same authors that demonstrated positive effects of whey protein hydrolysates (25 g) on promoting recovery of tissue damage (assessed via fibroblast proliferation) following a muscle damaging protocol in young adults [4]. Interestingly, in the study by Dale et al., (2015) different methods of preparing hydrolysates from the same source resulted in different in vitro and in vivo effects on fibroblast proliferation and repair of skeletal muscle damage, highlighting the importance of the processing method on the desired outcome(s) by mediating differences in specific peptides generated by the hydrolytic process [4]. However, it is worthy of note that the evidence for benefits of supplemental protein for enhancing acute exercise recovery, at least in the face of adequate dietary protein intake, irrespective of the specific source is equivocal [151, 152]. Nonetheless, protein intake was not reported in these studies [4, 149]. In a chronic study that combined daily ingestion of protein with 12 weeks whole-body resistance training, Mobley et al., (2017) found that both supplemental groups (whey protein concentrate and whey protein hydrolysate standardized to ~ 3.0 g of leucine per serving) exhibited similar training volumes and experienced statistically similar increases in strength, muscle thickness and total body skeletal muscle mass (as determined by dual X-ray absorptiometry; + 2.2 kg) compared with a maltodextrin control in untrained young males [150]. Further, type I and II fiber cross-sectional area increases were statistically similar across all supplemental conditions [150]. Similarly, there was a training, but no supplementation, effect on adipocyte cross-sectional area and no differences were found in satellite cell counts between the whey protein concentrate and hydrolysate [150]. However, it is worthy of note that the whey hydrolysate used in this study was only partially hydrolysed (12.5% degree of hydrolysis), yielding ~ 67% of peptides as < 5 kDa [150] and all groups consumed a minimum of 1.1 g·kg^−1^·day^−1^ of protein which further increased to ~ 1.3 g·kg^−1^·day^−1^ throughout the 12 weeks (assessed via self-reported nutritional intakes) [150].

In young resistance-trained men, following 8 weeks of resistance exercise training and 2 × 30 g/daily ingestion of whey protein hydrolysate, no differences in the improvements in lean mass and muscle strength were observed between a whey protein isolate and high-lactoferrin-containing whey protein concentrate (which is more comparable with a whey protein hydrolysate) [153]. In addition, no differences in serum markers of metabolic health (i.e., glucose, cholesterol, triglycerides), metabolism (i.e., urea nitrogen, creatinine) or muscle damage (i.e., creatine kinase) were found, with the exception of lower blood urea nitrogen with whey protein hydrolysate supplementation [153]. Based on the latter observation, despite observing no differences in muscle mass between proteins, the authors speculated that whey protein hydrolysate supplementation may improve metabolic efficiency by increasing carbohydrate and fat metabolism as well as decreasing protein catabolism compared with non-hydrolysed whey protein concentrates [115, 153]. Other studies have utilised whey protein hydrolysates to demonstrate positive effects of protein supplementation when combined with chronic exercise training programmes in recreational bodybuilders compared with a casein protein [154], elite soccer players compared with a maltodextrin control [155], and in young healthy females 4-days following a repeated-sprint exercise compared with an isoenergetic carbohydrate gel [156]. In addition, some contrasting findings have been observed investigating the performance and recovery effects of novel protein blends such as fish hydrolysates [157–160]. However, without appropriate protein controls, limited conclusions can be drawn from the aforementioned observations on the effectiveness of protein hydrolysates, per se. There is also evidence supporting the use of whey protein hydrolysates co-ingested with carbohydrate for increasing satellite cell proliferation [161] and muscle anabolic signaling [162], decreasing systemic markers of muscle damage [155, 163], accelerating recovery of functional performance [163, 164] and augmenting tendon and muscle hypertrophy, independent of resistance exercise contraction mode [165]. By contrast, a recent study found no impact of whey hydrolysate (~ 33 g) combined with carbohydrate on recovery of muscle function following resistance exercise in resistance-trained males compared with milk-based and flavoured-dextrose drinks [166]. However, as aforementioned, it is pertinent to note that carbohydrate co-ingestion with protein delays protein absorption and digestion kinetics [167].

In summary, the current available literature on the potential of protein hydrolysates to enhance exercise-induced adaptation and recovery is ambiguous, largely as a consequence of suboptimal designs in terms of inclusion of intact parent proteins as an appropriate comparator. To our knowledge there are currently no studies that have investigated the longer-term anabolic impact of other protein hydrolysates other than whey protein. Finally, whether supplementation with protein hydrolysates during recovery from intense exercise is associated with enhanced indices of training quality/quantity in subsequent sessions and thus, indirect favourable outcomes on longer-term muscle adaptive remodeling, are largely unknown.

## Considerations

Throughout this review, we have considered limitations with the current research on protein hydrolysates as well as proposed some of its possible applications and potential for future research. In the following section we provide a summary of; experimental considerations, future directions and practical applications of research into protein hydrolysates.

### Experimental considerations

In addition to the lack of appropriate comparator protein controls and the limitation(s) of the use of plasma AA concentrations as a means to infer differences in digestion and absorption kinetics, an interesting avenue for considering the potential of protein hydrolysates may be around their consumption within relative proximity to exercise [31]. Indeed, the consumption of slower digested proteins or consumption of larger quantities of fat and/or carbohydrates (as is observed with whole-foods), which would slow gastric emptying and absorption of proteins, may reduce the rates of MPS during postexercise recovery [33]. If a threshold of leucine needs to be met/exceeded to stimulate maximal MPS [35], then this may serve as an interesting application for hydrolysates to provide a muscle anabolic advantage. Furthermore, as discussed above, it is important to consider the relative dose of protein bolus, EAA and leucine content as well as the target population, as these factors are known to influence protein digestion and absorption and muscle anabolism [2, 33]. Further, the postprandial MPS response is not only determined by acute nutrient intake but is also likely modulated by habitual energy and nutrient intake and non-dietary factors such as, body composition, age, habitual physical activity, and/or sex [2, 33], and these are important considerations for interpreting the potential effects of protein hydrolysates and personalised nutritional recommendations. Consideration should be placed around the specific type of protein source as, for example, independent of the effects of hydrolysis, calcium caseinate is digested and absorbed more slowly when compared with micellar casein [44]. This highlights the importance of studies investigating protein sources (and particularly casein) in reporting the specific forms of the protein source as this adds further difficulty to the interpretations that can be drawn. Reporting and consideration of the storage of the protein may also represent an important consideration as it is possible that the storage of proteins may impact it’s digestive and absorption properties [44, 45]. As the digestion of AAs would be expected to be more rapid on an empty stomach and without the co-ingestion of other macronutrients [96, 101], fasted state studies could be further problematic in informing potential differences in digestion and absorption characteristics by increasing de novo synthesis of AAs particularly within the splanchnic region [81, 82]. Finally, whilst beyond the scope of this review, it is imperative to note that there is an inter-individual variability in response to nutrient (notably carbohydrate) digestion that is thought to be mediated by the microbiome [168], the role of which should not be overlooked within the context of protein research.

### Future directions and practical applications

There are currently limited studies directly comparing the effects of protein hydrolysates and respective intact proteins on longer-term skeletal muscle remodeling and health effects in a diverse range of populations. Further, there is a near complete absence of studies investigating lower quality plant-derived protein hydrolysates, which might provide an interesting platform for further research. Therefore, future research should continue to assess the impact of plant-proteins and novel protein blends (i.e., fish-derived proteins [169]) on skeletal muscle anabolic outcomes and metabolic health, without compromising appetite and subsequent energy (and protein) intake. It is imperative that future studies explore the potential differences of protein production of different sources (animal and plant-derived) and by comparison with appropriate protein controls particularly within relative proximity of exercise.

For a healthy young individual engaged in regular structured exercise training and consuming sufficient amounts of protein, it is unlikely based on the current available evidence that the exercise-induced muscle remodeling response would differ significantly between high-quality proteins of different manufacturing processes. Although speculative at this point, given the proposed mechanisms of protein hydrolysates on physiological outcomes discussed throughout (see Fig. 1), ingestion of a more rapidly digested protein hydrolysate may be more beneficial when insufficient per meal quantities of high-quality protein are consumed, or in populations where anabolic resistance to protein ingestion may be present (e.g., older adults) [70]. It is important to note that we do not advocate the replacement of protein-rich whole foods for supplemental proteins as a primary strategy to enhance an individual’s dietary protein intake. However, whilst the AA digestion/absorption kinetics (of different protein sources) and anabolic effects of protein hydrolysates co-ingested with other macronutrients warrants further attention, one might question whether providing protein-dense whole-foods is desirable in older or clinical populations considering their prolonged satiating effect and relatively slow rate of gastric emptying [170, 171], which may negatively affect overall daily dietary protein intake in populations prone to protein malnourishment. Instead, we recommend the use of isolated proteins as a supplement, as required (i.e., under conditions of insufficient protein intake). Whilst we acknowledge, unlike isolated proteins, protein rich whole-foods contain other non-protein derived nutrients that may further facilitate intramuscular anabolic signaling, MPS and tissue remodeling [172, 173], food fortification techniques may be an interesting application for protein hydrolysates that provide more di-, tri- and oligo-peptides, particularly in compromised patient settings.

## Data Availability

Not applicable.

## Abbreviations

AA: Amino acid
ACE: Angiotensin I-converting enzyme
BCAA: Branched-chain amino acid
DIASS: Digestible Indispensable Amino Acid Score
EAA: Essential amino acid
MPS: Muscle protein synthesis
mTORC1: Mechanistic Target of Rapamycin Complex1
PDCAAS: Protein Digestibility-corrected Amino Acid Score
